# Temperature Dependence of the Rayleigh Brillouin Spectrum Linewidth in Air and Nitrogen

**DOI:** 10.3390/s17071503

**Published:** 2017-06-26

**Authors:** Kun Liang, Jiaqi Xu, Peng Zhang, Yuanqing Wang, Qunjie Niu, Li Peng, Bo Zhou

**Affiliations:** 1 School of Electronic Information and Communications, Huazhong University of Science and Technology, Wuhan 430074, China; liangkun@hust.edu.cn (K.L.); jiaqixu@hust.edu.cn (J.X.); zhangpeng901@hust.edu.cn (P.Z.); eicnqj@hust.edu.cn (Q.N.); pengli@hust.edu.cn (L.P.); 2 Jiangsu Key Laboratory of Meteorological Observation and Information Processing, Nanjing University of Information Science and Technology, Nanjing 210044, China; 3 Laser Centre, Vrije Universiteit (VU), De Boelelaan 1081, 1081 HV Amsterdam, The Netherlands; yuanqing.wang@vu.nl

**Keywords:** atmospheric and oceanic optics, remote sensing and sensors, Lidar, atmospheric scattering, scattering measurements

## Abstract

The relation between spontaneous Rayleigh Brillouin (SRB) spectrum linewidth, gas temperature, and pressure are analyzed at the temperature range from 220 to 340 K and the pressure range from 0.1 to 1 bar, covering the stratosphere and troposphere relevant for the Earth’s atmosphere and for atmospheric Lidar missions. Based on the analysis, a model retrieving gas temperature from directly measured linewidth is established and the accuracy limitations are estimated. Furthermore, some experimental data of air and nitrogen are used to verify the accuracy of the model. As the results show, the retrieved temperature shows good agreement with the reference temperature, and the absolute difference is less than 3 K, which indicates that this method provides a fruitful tool in satellite retrieval to extract the gaseous properties of atmospheres on-line by directly measuring the SRB spectrum linewidth.

## 1. Introduction

The vertical profile of the atmospheric temperature for the stratosphere and troposphere (covering the altitude range from 0 to 30 km) plays an important role in meteorology, climatology, environmental protection, and space science. To measure the temperature, the Lidar technique, an advanced remote sensing tool, has been used. As the echo signal intensity is disturbed by Mie scattering caused by aerosols in the troposphere, the Lidar technique is applied, based on frequency field detection methods such as rotational Raman Lidar [1,2] and Rayleigh Brillouin (RB) Lidar. These techniques function by resolving the spontaneous Rayleigh Brillouin (SRB) spectrum [3,4] to reduce the Mie scattering. The rotational Raman differential backscattering cross-section is smaller than that of Rayleigh scattering, which means that the rotational Raman Lidar need a more powerful laser, a more sensitive receiver system, and more integration time. Therefore, the RB Lidar is more advantageous for tropospheric temperature measurements.

The RB technique, that works by resolving the SRB spectrum, is widely used in optical fiber [5,6] and seawater remote sensing [7,8,9]. However, this technique has recently been developed for atmospheric remote sensing applications, as the Rayleigh and Brillouin peaks (including Stokes and anti-Stokes) overlap considerably, making it more difficult to obtain the separate Rayleigh spectrum and Brillouin spectrum, which in turn makes it hard to derive the actual value of the SRB spectrum parameters from composite scattering profiles.

In order to describe the SRB spectrum, Tenti et al. established an S6 model [10] based on the Wang-Chang-Uhlenbeck equation [11] to analyze the spontaneous Rayleigh Brillouin scattering line shape of a molecular gas [12,13]. This model describes the SRB scattering line shape based on collision integrals in terms of the macroscopic transport coefficients of the gas as shear viscosity, bulk viscosity, thermal conductivity, and internal specific heat capacity per molecule. Therefore, it is possible that a parameter can be calculated using this model when the other parameters are known. Moreover, its good agreement with the experimental results in H_2_ (hydrogen), HD (hydrogen deuteride) and D_2_ (deuterium) was reported [14,15,16]. The S6 model can be used for monatomic gases as well, simply bysuppressing the internal degrees of freedom. Gu et al. [17,18] calculated temperature and bulk viscosity in this way. In 2014, the calculated (Tenti S6) line shapes were consistent with the experimental data at the residual level of 2% under laboratory conditions [17]. Also in 2014, the experimental data demonstrated that temperatures can be retrieved from SRB line shapes at an accuracy of 2 K based on Tenti S6 [18]. Furthermore, Witschas showed temperature profiles from 2 to 15.3 km derived from the SRB spectrum in 2015 [4]. The temperature measurements performed at noon showed good agreement to the radiosonde measurements. In fact, the temperature difference reached up to 5 K below the boundary layer and was smaller than 2.5 K above the boundary layer.

However, since the Tenti S6 is not represented in analytical form, it is mathematically involved and time consuming, which makes it not suitable for the real rapid remote sensing environment. Therefore, a better method aimed to establish the analytical relationship between the measured gas properties and spontaneous Rayleigh Brillouin scattering (SRBS) spectral characteristics such as those in fiber and ocean remote sensing applications. In gas application, it is important to obtain the spectrum characteristics such as the Brillouin shift and linewidth from the superposing SRBS spectrum. To solve this problem, models consisting of 3 Gaussian (G3) or 3 pseudo-Voigt (V3) functions have been proposed by Witschas [19,20] and Ma [21]. These models are analytical and benefit from rapid data fitting, through which the SRB spectrum characteristics can be obtained. However, the retrieval temperature based on the whole SRB line shape and Brillouin shift using the analytical model leads to discrepancies in the reference temperature up to 9.9 K [18,22]. So, these models need further improvement or a new retrieval method needs to be proposed based on other spectrum characters.

**Figure 1:**
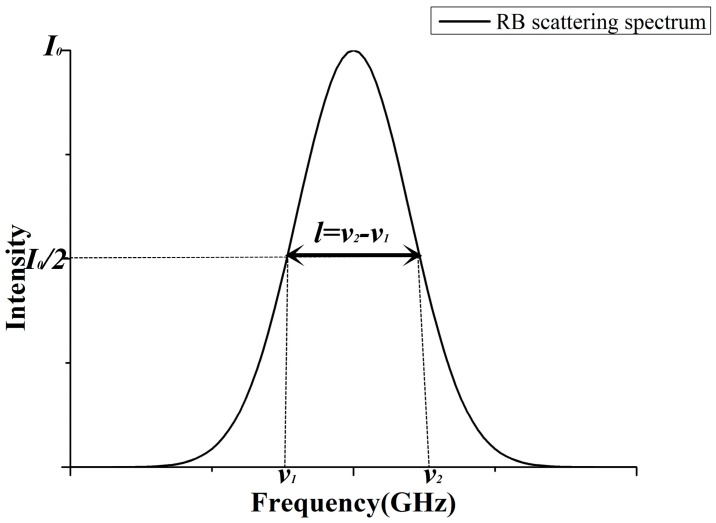
The diagram of the whole spectrum linewidth (full width at half height, FWHH) in an spontaneous Rayleigh Brillouin (SRB) scattering spectrum.

In satellite retrieval, such as the The Atmospheric Dynamics Mission (ADM)-Aeolus satellite mission initiated by European Space Agency (ESA) [23], when extracting the gaseous properties of atmospheres on-line, the whole spectrum linewidth (full width at half height, FWHH, as shown in Figure 1) is the only information directly obtained from the overlapped SRB spectrum. Therefore, utilizing the dependence of the linewidth of the SRB spectrum on temperature is a direct and effective method for the remote sensing of the atmosphere temperature. In previous studies [24], it was assumed that the linewidth is proportional to the square root of the atmospheric temperature. However, this assumption is not accurate because the dependence is not strictly linear. In addition, the atmosphere pressure will result in molecular collision, which results in the linewidth being different at the same temperature but with a different pressure. Therefore, the purpose of this paper is to analyze, for the first time, the dependence of the linewidth of the SRB spectrum on temperature. Furthermore, a method for retrieving atmospheric temperature based on the temperature dependence of the SRB spectrum linewidth is proposed. This method is of major importance for the remote sensing of atmospheric temperature with a serious advantage as it is an analytic model, and constitutes a useful tool for satellite retrieval.

## 2. Temperature Dependence of the Rayleigh Brillouin Spectrum Linewidth

In theory, the linewidth of the SRB spectrum has a strong dependence on temperature, and a model has been built to explain this relationship between the linewidth and temperature. However, so far no detailed studies have demonstrated this relationship. The Tenti S6 model, which is considered as the best model for describing SRB spectra of light scattered in molecular gases of single species, can be applied to aid in analyzing the relationship, but it is difficult to use it to retrieve temperature directly because it is nonanalytic. The SRB spectrum could be calculated by Tenti S6 model at different values of temperature and pressure, and the relationship between the linewidth and temperature can be analyzed through an optimization procedure as a least-squares fit with temperature and linewidth.

Considering the Lidar application, the temperature *T* should be regarded as a dependent variable, while the linewidth *l* (directly obtained from the SRB spectrum) and the pressure (acquired by 1976 U.S. Standard Atmosphere Model [25]) should be independent variables. Through the Tenti S6 model, a model based on the dependence of the linewidth of the SRB spectrum on temperature and pressure can be built to calculate the atmospheric temperature.

While the main purpose of this paper is to discuss this relationship between mono-molecular nitrogen and air in atmospheric pressure and temperature conditions, values for the linewidth are calculated for the wavelengths at 403 nm and 366 nm.

According to the 1976 U.S. Standard Atmosphere Model [25], under the typical climate in the northern hemisphere, and in the lower atmosphere (0 to 15 km) of the vertical height, the range of atmospheric temperature is about 220–340 K and the range of atmospheric pressure is about 0.1–1 bar. We calculate the SRB spectrum linewidth at the temperature and pressure using the S6 model, the gas transport coefficients of which are shown in Table 1, for N_2_ and air. Then, a data fitting procedure is implemented to find a numerical solution.

Here, the characteristic constants for air are as follows: *η_0_* = 1.716 × 10^−5^ kg·m^−1^·s^−1^ is the reference shear viscosity and *k_0_* = 24.1 × 10^−3^ W·m^−1^·K^−1^ is the reference thermal conductivity at reference temperature *T*_0_ = 273 K, *T_η_* = 111 K, *T_k_* = 194 K. As for N_2_, *η_0_* = 1.663 × 10^−5^ kg·m^−1^·s^−1^ and *k_0_* = 24.2 × 10^−3^ W·m^−1^·K^−1^ at reference temperature *T*_0_ = 273 K, *T_η_* = 107 K, *T_k_* = 150 K are the characteristic constants [28].

**Figure 2:**
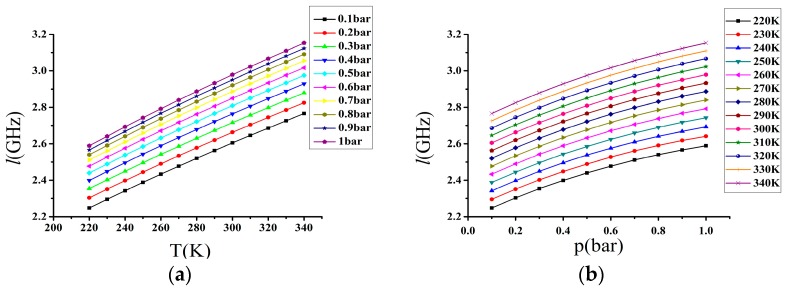
(**a**) The relation between the linewidth and temperature with fixed pressure for N_2_; (**b**) The relation between the linewidth and pressure with fixed temperature for N_2_.

Taking temperature and pressure as two independent variables, we obtain each value of the linewidth, which are listed in Table 2. As shown in Figure 2, the values of the linewidth range from 2.2 to 3.2 GHz. At the same temperature with the pressure from 0.1 to 1 bar, the linewidth changes about 340 MHz, which means that the linewidth is not only influenced by the temperature, but also the pressure. In addition, the relation between the linewidth and temperature is nearly linear, but the relation between the linewidth and pressure is not, as the change in the linewidth between 0.1 and 0.2 bar is about 57 MHz, but the change between 0.9 and 1.0 bar is only about 27 MHz at the same temperature. As the temperature increases by 10 K, the linewidth linearly rises about 40 MHz at the same pressure.

Taking the 130 sets of linewidth (*l*), temperature (*T*), and pressure (*p*), a least-squares fit is used to determine a function *T*(*l,p*) of the form as:

(1) $$T(l,p)=c_{0}+c_{1}l+c_{2}p+c_{3}l^{2}+c_{4}p^{2}+c_{5}lp+c_{6}l^{3}+c_{7}p^{3}+c_{8}lp^{2}+c_{9}l^{2}p$$

The resulting coefficients in the fitting expression for *T*(*l,p*) for N_2_ are given in Table 3, and those for air are given in Table 4.

**Figure 3:**
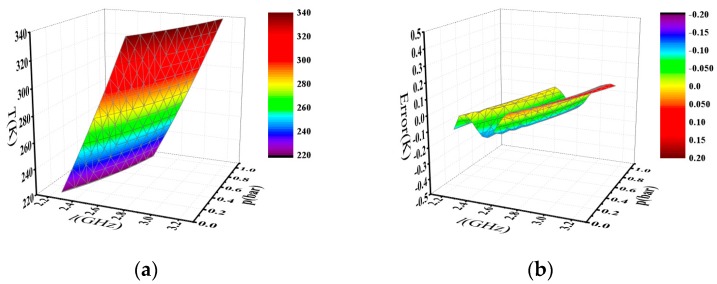
(**a**) The relation between the temperature, linewidth, and pressure for N_2_; (**b**) Difference between the temperature calculated by Equation (1) and the corresponding temperature value listed in Table 1 for N_2_.

The retrieval temperature based on Equation (1) from the SRB spectrum linewidth and pressure is shown in Figure 3a. The difference between *T*(*l,p*) from Equation (1) and the corresponding value of *T* from Table 2 is shown in Figure 3b, which describes that the max deviation is about 0.15 K and the standard deviation is 0.07 K. As for air, the max deviation is about 0.21 K and the standard deviation is 0.07 K. Therefore, Equation (1) is consistent with the data in Table 2.

Because we have taken pressure as one independent variable in Equation (1), the pressure needs to be known when the temperature is retrieved from the linewidth. We assume that knowledge of the pressure at one height and location can be calculated from the 1976 U.S. Standard Atmosphere Model and the barometric height formula with only 1% error [29].

The changes of temperature, which are caused by small changes in the linewidth and pressure, represent the sensitivity of the retrieval model. These values of the changes enable us to determine the limit on the accuracy of temperature retrieving based on the measurements of the linewidth and assuming knowledge of the pressure. Thus, the dependence of the temperature on one variable while fixing the other variable is expressed by:

(2) $$\frac{\partial T}{\partial l}=c_{1}+2c_{3}l+c_{5}lp+3c_{6}l^{2}+c_{8}lp^{2}+2c_{9}lp$$

(3) $$\frac{\partial T}{\partial p}=c_{2}+2c_{4}p+c_{5}l+3c_{7}p^{2}+2c_{8}lp+c_{9}l^{2}$$

**Figure 4:**
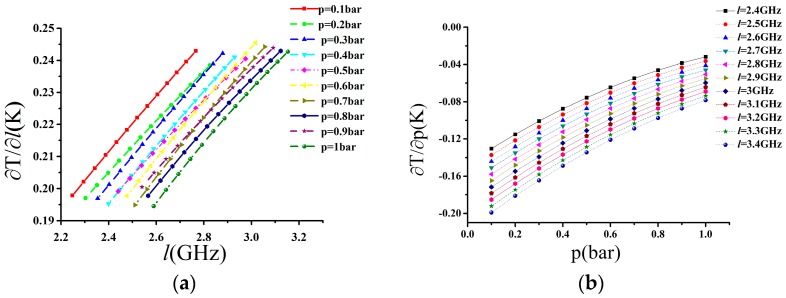
(**a**): Uncertainty of temperature plotted as a function of the linewidth standard deviation for 1 MHz; (**b**): Uncertainty of temperature plotted as a function of the pressure standard deviation for 1 mbar.

In Figure 4a, the uncertainty of temperature is plotted as a function of the linewidth standard deviation for 1 MHz, and in Figure 4b, the uncertainty of temperature is plotted as a function of the pressure standard deviation for 1 mbar.

Based on recent studies on high spectral resolution Lidar systems, we consider a typical condition of 2 km altitude in the standard atmosphere; the uncertainty of the SRB spectrum linewidth measurement is *Δl* = 10 MHz, and the corresponding uncertainty of temperature is about 2.3 K. The relative error of pressure is about 1% based on the barometric height formula, which result in an uncertainty of temperature value of 0.07 K. The root mean square error in measurements of the temperature uncertainty can be expressed by:

(4) $$\Delta T={\left[{\left(\frac{\partial T}{\partial l}\right)}^{2}{\left(\Delta l\right)}^{2}+{\left(\frac{\partial T}{\partial p}\right)}^{2}{\left(\Delta p\right)}^{2}\right]}^{1/2}$$

From Equation (4), the limit on the accuracy in the retrieval of temperature can be calculated, and the uncertainty of temperature is 2.3 K. The accuracy is acceptable for real remote sensing applications.

## 3. Experimental Results and Discussion

To further evaluate the performance of this method, some experimental data is used to analyze the accuracy of retrieval temperature. In this paper, the SRB spectrum linewidth for N_2_ is derived from spontaneous RB-scattering experiments at 403 nm for a range of pressure. The detailed experimental setup is described in Reference [17]. The laser beam with an effective power of 5 W at 403 nm crosses the gas cell, after which the scattered light is collected at 90° as an uncertainty of 0.9° with respect to the beam direction. Then, the scattered light is spectrally filtered by a Fabry-Perot Interferometer (FPI) with an instrument linewidth of 128 MHz and a free spectral range of 7478 MHz, and finally is recorded by Photomultiplier tubes (PMT) and outputted to a computer.

Experimentally, SRB scattering in N_2_ was measured on a baratron, in which the pressure value can be measured with an accuracy of 0.15%. The temperature of the N_2_ was measured with two Pt100 thermo-resistors mounted on the top and bottom of the gas cell, delivering an accuracy of about ±0.25 K. The gas cell with a water cooling system, which can be used both a cooler and heater, allows for a temperature variation of the gas sample from 240 to 340 K. The SRB scattering spectrums we used were measured in N_2_ at different pressures from 0.1 to 1 bar.

Assuming that the measured pressure is accurate in the experiments, the uncertainty of pressure can be neglected. Thus, the temperature uncertainty is derived from the uncertainty of the SRB spectrum linewidth measurements, which are mainly caused by the noise on the measured data points and the uncertainty of scattering angles. The noise contribution is estimated by using the Poisson function, which can be determined with a standard deviation *Δl_noise_* = *l* × (*N*)^1/2^, where *l* is the linewidth which is between 2.3 and 3.4 GHz, and *N* is the number of detected photons which is about 10^6^. Therefore, the uncertainty caused by the noise is about 2.3–3.4 MHz. By using the Tenti S6 model, the corresponding linewidth uncertainty is about 17.9–23.3 MHz when the scattering angle is 90° with an uncertainty of 0.9°. The root-mean-square error (uncertainty) in measurements of the linewidth can be expressed in terms of the uncertainties *Δl_noise_* and *Δl_angle_*:

(5) $$\Delta l={\left[\Delta l_{noise}^{2}+\Delta l_{angle}^{2}\right]}^{1/2}$$

Therefore, the linewidth uncertainty is about 18.1–23.5 MHz, which leads to a temperature uncertainty of about 5–6 K. However, it must be mentioned that the estimate of the temperature uncertainty is quite macroscopic and gives the maximum possibility. Considering that the laser beam was only slightly re-aligned between each measurement, we use the Tenti S6 model to analyze the scattering angle measurement error in Table 5. The results demonstrate that the error is much smaller than this estimation. As for the Lidar measurements in reality, which are usually restricted to a scattering angle of 180° and a small field of view, the scattering angle uncertainty only plays a minor role in temperature retrieval from RB profiles.

The temperature *T_model_* we retrieved from the RB spectrum linewidth *l*_measure_ based on the experimental data is compared with the temperature *T_Pt100_* measured by Pt100, which is used as a reference. Meanwhile, we calculate the Tenti S6 model spectrum linewidth *l*_theory_ using *T_Pt100_* and the pressure in the experimental condition, through which the performance of this method can be evaluated. In fact, the spectrum obtained using FPI provides a convolution spectrum based on interference rather than a direct scattering spectrum, which can be expressed as [18]:

(6) $$I=(I_{RBS}(v)+I_{par}\;\times \delta_{par}(v))⊗f(v)$$

where ⊗ denotes a convolution, *v* is the optical frequency, *I_RBS_*(*v*) is the spectral distribution of SRB scattering, *I_par_ × δ_par_*(*v*) is the light scattered on particles or spurious reflection [18], and *f*(*v*) is the instrument function with a linewidth of 128 MHz.

**Figure 5:**
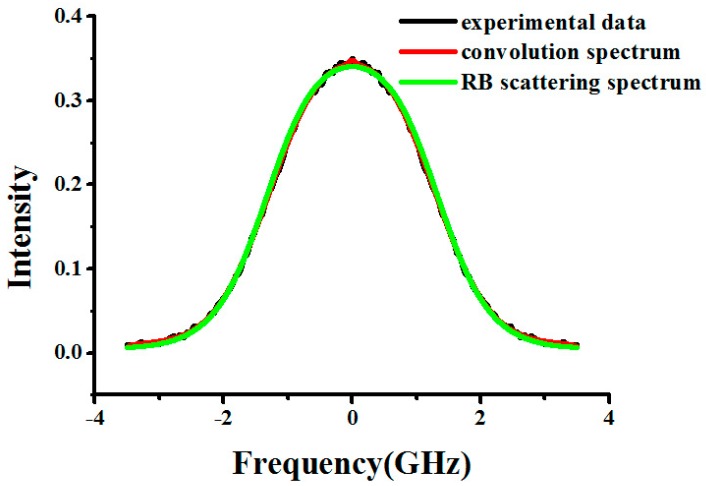
The experimental data (black line), convolution spectrum (red line), and SRB scattering spectrum (green line) when pressure is 1.091 bar and temperature is 297.4 K for N_2_ at 403 nm.

**Figure 6:**
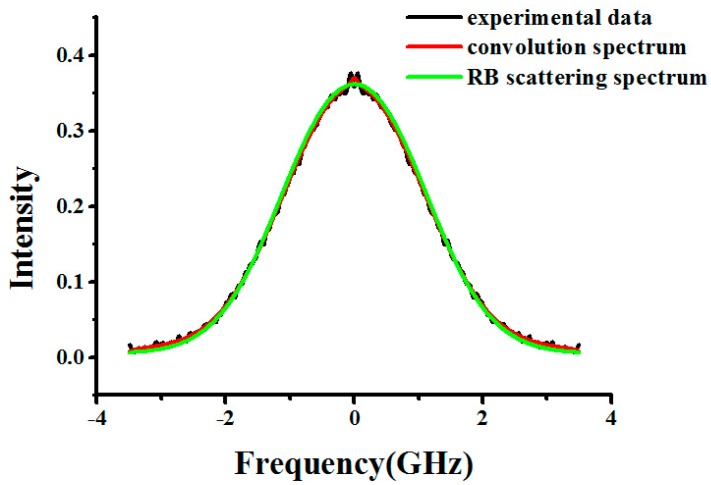
The experimental data (black line), convolution spectrum (red line), and SRB scattering spectrum (green line) when pressure is 0.108 bar and temperature is 295.3 K for N_2_ at 403 nm.

The S6 model should not be used to obtain the spectrum because the temperature should be unknown. Here, the analysis model Equation (6) is used to describe the SRB spectrum and measure the linewidth. The experimental data and the best-fit spectrum are shown in Figure 5 and Figure 6, and it can be seen that the light scattered on particles or spurious reflection is obvious and that Equation (6) is helpful for removing the effect of this scattering. Under the experimental conditions, the retrieved results are shown in Table 6. In this paper, particle scattering can be described by a Dirac-delta function due to the fact that the particle scattering spectral broadening is negligible. However, in some cases, the contribution of particle scattering to the signal from SRB scattering is much larger, especially in real atmospheric measurements, where the amount of particle scattering (e.g., from the aerosol-rich boundary layer or sub-visible cirrus clouds) can be much larger. In the future, we will employ atmospheric high-spectral resolution Lidar to investigate the effect of light scattering on particles and aerosols in the SRB scattering spectrum in order to find a better solution for this issue.

**Figure 7:**
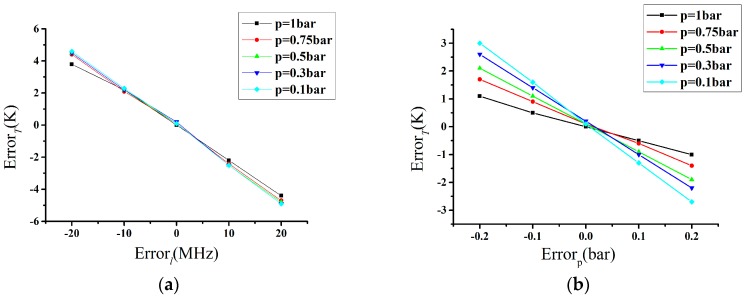
(**a**) The influence of linewidth measurement error on retrieved temperature; (**b**) The influence of pressure measurement error on retrieved temperature.

In order to quantitatively evaluate the performance of the temperature retrieval model, we can obtain the corresponding temperature error by changing the pressure and linewidth step by step. As shown in Figure 7, the effect of the pressure measurement error on temperature is far less than the linewidth measurement error. Therefore, in practical application when using the US standard atmospheric model to provide the pressure, the temperature is available on the basis of the relationship between the SRBS linewidth and temperature. Meanwhile, we also analyze the linewidth error, which is mainly caused by the error of the scattering angle. On the one hand, using the Tenti S6 model to fit the experimental data of N_2_ at 403 nm, we can obtain the scattering angle *θ*^’^ and calculate the corresponding linewidth *l*^’^ and temperature *T*^’^ though this scattering angle *θ*^’^ [30]. On the other hand, the angle *θ* was set in Equation (1) and is assumed to be 90°. As we can see from the results in Table 6, the error of the scattering angle is less than 0.6°, which means that the error of the temperature will not exceed 3 K. We adopt the Tenti S6 model using the fixed bulk viscosity values to obtain the relation between the temperature and linewidth; however, some research has shown that the bulk viscosity values are temperature-dependent [31], which results in certain temperature differences for the Equation (1). Therefore, we calculate the linewidth deviation between the fixed bulk viscosity and the bulk viscosity dependent on temperature in Reference [31], and the results show that the deviation of the linewidth is less than 10 MHz at the range from 260 to 340 K, which brings the temperature deviation to less than 2 K.

While the main purpose of the present study is to verify the proposed model for N_2_ and air in atmospheric pressure and temperature conditions, values for the temperature are derived for wavelength 366 nm using the data in a previous study measuring SRB-scattering in air [30]. The retrieved results are shown in Table 7.

There is also very good agreement between temperatures retrieved with the linewidth and reference temperature in air. In particular, the absolute difference is less than 3 K for all measurements.

In real remote sensing applications, the scattering angles are 180° for the received signal after a long-distance transmission. In such cases, the uncertainty of the scattering angles can be ignored, that is, the temperature uncertainty is caused by the uncertainty in SRB spectrum linewidth measurements, which are mainly caused by the noise.

The results shown above demonstrate that temperatures can be derived from the obtained RB spectrum linewidth in N_2_ and air with an accuracy of results within 3 K, which is acceptable for real remote sensing applications. Though the accuracy is lower than that based on the Tenti S6 model, this proposed method has the advantages of directly measuring the SRB spectrum linewidth and faster processing, which constitutes a useful tool for satellite retrieval and extracting the gaseous properties of atmospheres on-line.

## 4. Conclusions

In this paper, the dependence of the SRB spectrum linewidth on atmospheric temperature and pressure is analyzed, a model retrieving atmospheric temperature from the directly measured linewidth is established, and the uncertainty of temperature is estimated. The temperature in this model is only calculated by two independent variables. One is the linewidth, which can be directly measured from the obtained SRB spectrum. The other is the pressure, which can be obtained from the 1976 U.S. Standard Atmosphere Model. Two groups of experimental data at different pressures ranging from 0.1 bar to 1 bar and different temperatures ranging from 240 K to 340 K are used to verify the performance of the proposed method. The results show that the absolute difference between the derived temperature and reference temperature is less than 3 K for measuring temperature in the atmosphere, which proves that the proposed method is simplified and suitable for the remote sensing of tropospheric temperature.

## Figures and Tables

**Table 1:** Gas transport coefficients for N_2_ and air used for Tenti S6 model calculations.

Gas		N_2_	Air
Mass number	[g·mol^−1^]	28	28.970
Bulk viscosity *η_b_*	[kg·m^−1^·s^−1^]	1.290 × 10^−5^ [26]	1.108 × 10^−5^ [26]
Shear viscosity *η*	[kg·m^−1^·s^−1^]	$\eta =\eta_{0}{\left(\frac{T}{T_{0}}\right)}^{3/2}\times \frac{T_{0}+T_{\eta }}{T+T_{\eta }}$ [27]	
Thermal conductivity *k*	[W·m^−1^·K^−1^]	$k=k_{0}{\left(\frac{T}{T_{0}}\right)}^{3/2}\times \frac{T_{0}+T_{k}}{T+T_{k}}$	
Heat capacity ratio *γ*		1.4	1.4
Internal specific heat *c_int_*		1.0	1.0

**Table 2:** SRB spectrum linewidth (GHz) with various temperatures and pressures for N_2_ at 403 nm.

*p* (bar)	*T* (K)												
	220	230	240	250	260	270	280	290	300	310	320	330	340
0.1	2.248	2.295	2.342	2.388	2.433	2.477	2.520	2.563	2.605	2.646	2.686	2.726	2.766
0.2	2.303	2.351	2.398	2.444	2.490	2.534	2.578	2.620	2.663	2.704	2.745	2.785	2.825
0.3	2.354	2.402	2.449	2.496	2.542	2.586	2.630	2.673	2.716	2.757	2.798	2.839	2.879
0.4	2.399	2.448	2.496	2.543	2.589	2.634	2.678	2.722	2.764	2.806	2.848	2.888	2.929
0.5	2.440	2.489	2.538	2.585	2.632	2.677	2.722	2.766	2.809	2.851	2.893	2.934	2.975
0.6	2.477	2.527	2.576	2.624	2.671	2.717	2.762	2.806	2.850	2.892	2.934	2.976	3.017
0.7	2.511	2.561	2.610	2.659	2.706	2.753	2.798	2.843	2.887	2.930	2.972	3.014	3.056
0.8	2.540	2.591	2.641	2.690	2.738	2.785	2.831	2.876	2.921	2.964	3.007	3.049	3.091
0.9	2.566	2.618	2.668	2.718	2.767	2.814	2.861	2.906	2.951	2.995	3.039	3.081	3.123
1.0	2.589	2.641	2.693	2.743	2.792	2.840	2.887	2.933	2.979	3.023	3.067	3.110	3.153

**Table 3:** Coefficients in the fitting expression for Equation (1) for N_2_ at 403 nm.

Coefficient	Value	Coefficient	Value
c_0_	31.2793823337473	c_5_	−72.6433430799775
c_1_	−47.9509027189756	c_6_	−2.68227909475931
c_2_	25.9803346707134	c_7_	−18.2377554932758
c_3_	67.2537824011778	c_8_	12.2919823136653
c_4_	55.340098697962	c_9_	0.27046043741303

**Table 4:** Coefficients in the fitting expression for Equation (1) for air at 366 nm.

Coefficient	Value	Coefficient	Value
c_0_	136.127370368944	c_5_	−160.587794624564
c_1_	−178.869256282723	c_6_	−9.3473932415678
c_2_	54.0470103235185	c_7_	11.8503768580463
c_3_	110.814219167605	c_8_	−57.2742042466781
c_4_	193.641249446796	c_9_	33.1734261826669

**Table 5:** The error between the experimental data and the theoretical values caused by the scattering angle.

*p* (bar)	*θ*_(deg)_	*θ^’^*_(deg)_	*θ* − *θ*^’^_(deg)_	*L*_(GHz)_	*l*^’^_(GHz)_	*l* − *l*^’^_(MHz)_	*T*_Pt100__(K)_	*T*^’^_(K)_	*T*_Pt100_ − *T*^’^_(K)_
1.091	90	90.16	−0.16	2.990	2.993	−3	297.4	298.1	−0.7
0.749	90	90.28	−0.28	2.890	2.897	−7	296.9	298.4	−1.5
0.506	90	89.46	+0.54	2.801	2.790	+11	297.5	294.9	+2.6
0.313	90	90.47	−0.47	2.714	2.724	−10	297.9	300.4	−2.5
0.108	90	89.74	+0.26	2.591	2.585	+6	295.3	294.0	+1.3

**Table 6:** The results of the retrieved temperature under experimental conditions for N_2_ at 403 nm.

*p* (bar)	*l*_theory_ (GHz)	*l*_measure_ (GHz)	*l*_theory_ − *l*_measure_ (MHz) (MHz)	*T*_Pt100_ (K)	*T*_model_ (K)	*T*_Pt100_ − *T*_model_ (K)
1.091	2.990	2.990	0	297.4	297.4	0
0.749	2.890	2.885	+5	296.9	295.8	+1.1
0.506	2.801	2.788	+13	297.5	294.5	+3
0.313	2.714	2.714	0	297.9	298.0	−0.1
0.108	2.591	2.578	+13	295.3	292.4	+2.9

**Table 7:** The results for the retrieved temperature under experimental conditions for air at 366 nm.

	*p* (bar)	*l*_theory_ (GHz)	*l*_measure_ (GHz)	*l*_theory_ − *l*_measure_ (MHz)	*T*_Pt100_ (K)	*T*_model_ (K)	*T*_Pt100_ − *T*_model_ (K)
Air~1 bar	0.858	2.989	2.989	0	254.8	254.8	0
	0.947	3.110	3.121	−11	276.7	279.2	−2.5
	1.013	3.218	3.225	−7	297.3	299.5	−2.2
	1.013	3.304	3.309	−5	318.3	320	−1.7
	1.017	3.388	3.380	+8	337.8	337.6	+0.2
Air~0.75 bar	0.643	2.919	2.925	−6	254.8	256.3	−1.5
	0.703	3.035	3.032	+3	276.8	276.1	+0.7
	0.726	3.129	3.132	−3	297	298.1	−1.1
	0.776	3.229	3.224	+5	317.7	316.6	+1.1
	0.826	3.323	3.316	+7	337.2	335.8	+1.4
